# Epigenetic Characteristics in Primary and Recurrent Glioblastoma—Influence on the Clinical Course

**DOI:** 10.3390/biomedicines12092078

**Published:** 2024-09-12

**Authors:** Alexander Quiring, Hannah Spielmann, Fritz Teping, Safwan Saffour, Fatemeh Khafaji, Walter Schulz-Schaeffer, Nathan Monfroy, Joachim Oertel, Stefan Linsler, Christoph Sippl

**Affiliations:** 1 Department of Neurosurgery, Klinikum Rechts Der Isar, Technical University Munich School of Medicine, 80333 Munich, Germany; 2 Department of Neurosurgery, Faculty of Medicine, Saarland University, 66421 Homburg/Saar, Germany; 3 Department of Neurosurgery, Medical Campus Oberfranken of FAU Erlangen, 91054 Bayreuth, Germany; 4 Institute of Neuropathology, Faculty of Medicine, Saarland University, 66421 Homburg/Saar, Germany

**Keywords:** glioblastoma micro-RNA, epigenetic, methylation, recurrence

## Abstract

Objective: Epigenetic tumor characteristics are in focus for glioblastoma prognosis. This raises the question if these characteristics present with stable expression during the progression of the disease, and if potential temporal instability might influence their prognostic value. Methods: A total of 44 patients suffering from glioblastoma who were treated for their primary and relapse tumors were included in the study. Tumor specimens from the initial and recurrent tumor resection were subjected to evaluation of *MGMT*, *p15*, and *p16* methylation statuses. MiRNA-21, -24, -26a, and -181d expression was evaluated as well. The stability of these epigenetic markers during the progression of the disease was correlated with further clinical data. A Cancer Genome Atlas (TCGA) dataset of 224 glioblastoma patients was used as an independent cohort to validate the results. Results: Instability was observed in all examined epigenetic markers. *MGMT* methylation changed in 30% of patients, *p15* methylation changed in 35%, and *p16* methylation changed in 37.5% of cases. MiRNA expression in corresponding initial and relapse tumor specimens varied considerably in general, individual cases presented with a stable expression. Patients with a decreased expression of miRNA-21 in their recurrence tumor showed significantly longer overall survival. These results are supported by the data from TCGA indicating similar results. Conclusions: Epigenetic characteristics may change during the course of glioblastoma disease. This may influence the prognostic value of derived molecular markers.

## 1. Introduction

Glioblastoma multiforme is the most prevalent primary brain tumor [1]. This disease presents with an exceptionally aggressive progression and yields a dire prognosis, with a 5-year survival rate under 3% [2]. Elements such as age at diagnosis, extent of resection, Karnofsky performance score, Isocitrate dehydrogenase 1 (*IDH1*) mutation status, and the methylation status of the O6-methylguanine-DNA methyltransferase (*MGMT*) promoter have been identified as pivotal factors influencing individual prognoses [3,4,5,6]. The established therapeutic regimen encompasses surgery, radiation, and chemotherapy [7]. In recent years, an array of molecular markers, predominantly of an epigenetic nature, have emerged as candidates for prognostication and prediction of treatment responses. Among these markers, in addition to MGMT, the methylation status of *p15* and *p16* have garnered significant attention [8]. Functioning within the P15/P16/RBI/E2F pathway, *p15* and *p16* wield a critical influence over the cell cycle [9]. Alongside these, micro-RNAs (miRNAs) have become prospective tools for prognosticating disease progression [10]. MiRNAs, short noncoding RNA molecules, orchestrate gene expression suppression via post-transcriptional mechanisms [11]. A multitude of miRNAs have demonstrated the capacity to sway the prognostic landscape of glioblastoma (GBM) patients [12,13]. Notably, miRNA-21 exerts an oncogenic impact by impeding cellular apoptosis [14]. MiRNA-24 exerts its influence over the P15/P16/RB1/E2F pathway [15,16,17]. This pathway is crucial in cell cycle control and its dysregulation is a central aspect of gliomagenesis. Removal of the *miRNA-24* gene correlates with a deceleration in tumor proliferation by directly suppressing P16 [18]. MiRNA-26a governs *PTEN* and RB1 in the above-mentioned pathway [19,20], contributing to oncogenesis, tumor growth, and angiogenesis [21]. MiRNA-181d directly impacts *MGMT* and serves as a tumor suppressor through its modulation of *Bcl-2* and *K-Ras* [22].

With the majority of GBM patients experiencing relapse after a certain period, those maintaining a clinically acceptable condition necessitate a second-line therapy [23].

An unanswered query pertains to the stability of these aforementioned molecular epigenetic markers, and, consequently, their reliability over time and throughout disease progression. This applies not only to miRNAs but also to the promoter methylation of specific genes. Research has unveiled instances where *MGMT* promoter methylation exhibited instability between primary and recurrent GBM cases, occurring in 25–40% of instances [24,25].

Alterations in the stability of MGMT methylation over time have also demonstrated an impact on survival rates [26]. Notably, no studies have delved into this matter in relation to miRNAs, p15, and p16 methylation statuses. The present study endeavors to assess the temporal constancy of expression pertaining to the aforementioned markers within initial and recurrent tumor specimens. Furthermore, this investigation seeks to determine whether potential dynamics in the expression of these parameters bear an influence on disease prognosis.

## 2. Methods

### 2.1. Study Design and Selected Epigenetic Characteristics

Specific epigenetic characteristics were selected to estimate temporal stability due to their known impact on prognosis in GBM. The most widely studied are the methylation patterns of *MGMT*, *p15,* and *p16* [6,27,28]. Furthermore, miRNA-21, as the most actively studied miRNA in GBM as well as the miRNA-24, -26, and -181d, which have proven to impact prognosis and therapy response, were chosen [29,30,31,32].

To evaluate the stability of certain epigenetic characteristics over time, tumor specimens from initial GBM resection as well as recurrent tumor tissue from the same patients were used.

Epigenetic results of the initial surgery were compared to those of the recurrent surgery and correlated with corresponding clinical development. The methods used to assess methylation status and miRNA expression are described below. These findings, with a special focus on differences between initial and relapse tumors, were then descriptively highlighted and correlated with clinical outcome data and survival. An objective control cohort from a TCGA dataset was eventually correlated with the findings presented in this study. It has to be noted that the presented TCGA data only account for primary GBM samples since the databank does not contain data from corresponding primary and recurrent glioblastoma cases.

### 2.2. Patients

Tumor tissues from 40 patients with recurrent GBM were used in the present trial. All patients underwent surgery on their primary tumor and the recurrence at Saarland University Medical Center in Homburg, Germany between 2006 and 2020. Clinical data sets of all patients were available from 2006 to 2024. Inclusion criteria are neuropathologically approved diagnosis of GBM without prior history of a lower grade astrocytoma; sufficient tissue from initial and recurrent tumor for further analysis; and the informed consent of the patient. The specimens collected were graded using the current World Health Organization classification of brain tumors of 2021, including a wild type IDH1/2 status. All tumor samples were snap-frozen and stored at −80 °C before analysis. All patients were treated with consecutive concomitant radio-chemotherapy [7].

This study was approved by the local German ethical board (Ethikkommision der Ärztekammer des Saarlandes, Saarbrücken, Germany), and written informed consent was obtained from all patients (General Medical Council of the State of Saarland, NO 93/16).

### 2.3. Methylation Analysis

Methylation analysis of the promoter regions of *MGMT*, *p15*, and *p16* was conducted as previously described by using methylation-specific PCR [33]. Complete methylation analysis could, due to technical issues, only be achieved in 42 patients; 1 patient was lost with its initial and relapse tumor, another only the with its relapse specimen. This is highlighted further down in the graphics in red.

### 2.4. MiRNA Analysis

Isolation of miRNA from tumor specimens was performed using a miRNeasy miRNA Isolation kit (QIAGEN N.V., Venlo, the Netherlands) according to the manufacturer’s protocol. Evaluation of miRNA expression levels was conducted as previously described via quantitative reverse transcription–polymerase chain reaction (qRT-PCR) [29]. Primers for miR-21, -24, -26a, and -181d were purchased from Applied Biosystems (TaqMan MicroRNA Assay; miR-21, ID 000397; miR-24, ID 000402; miR-26a, ID 001848; miR-181d, ID 001023; Applied Biosystems). PCR was conducted in triplicate, and a negative control without a template was also tested. Quantitative miRNA expression data were calculated using the comparative cycle threshold (Ct) method with RNU48 as an accredited and stable reference miRNA. For miRNA expression, relative expression level (REL) was calculated as follows: REL _tumor_ = 2^− (CT-miRNA tumor − CT-RNU48 tumor)^. The expression (REL) of miRNA-21, -24, -26a, and -181d was normalized to RNU48.

### 2.5. Statistical Analysis

All statistical analyses were performed using SPSS, version 23 (IBM Corp., Armonk, New York, USA). A model of linear regression and multivariate linear regression was used to analyze association between miRNA expression, methylation status, and clinical parameters such as overall survival, progression-free survival, and age at disease onset. Disease progression was defined either radiologically as a new contrast dye enhancing tumor formation, death of the patient, or a reduction in the Karnofsky performance score of more than 30 points [34]. Cox regression was used to analyze overall survival and progression-free survival. A value of *p* < 0.05 was defined as statistically significant. A value of *p* < 0.10 was considered a statistical trend. To compare relative miRNA expression between initial and relapse tumor, Mann–Whitney U-test was used. The standard deviation is presented by ±. The range of expression is presented in squared brackets [].

Clinical correlation with differences in methylation status and miRNA expression between initial and relapse tumors was verified with an independent TCGA cohort, established using expression data from an Agilent Human miRNA_8 × 15 k array. The TCGA cohort consisted of 224 GBM cases. TCGA level 3 data regarding miRNA expression were retrieved using the Oncolnc platform (http://www.oncolnc.org, accessed on 1 June 2024) [35].

## 3. Results

### 3.1. General Results of the Study Population

Factors influencing survival were age at onset of the disease (*p* = 0.02) and initial Karnofsky performance score (*p* = 0.01), as well as the extent of the initial tumor resection (*p* = 0.04).

Further information on the study population can be found in Table 1.

### 3.2. Stability of Promoter Methylation

The *MGMT* promoter in the initial tumor specimen was methylated in 21 (52.5%) cases. Another 19 (47.5%) cases harbored no methylation at this site. The corresponding relapse tumors revealed 15 (37.5%) cases with and 24 (60%) cases without *MGMT* methylation. One patient was not to be evaluated due to technical issues. Whilst 27 (67.5%) patients of the total study population showed stable rates of *MGMT* promotor methylation, only 12 (30%) patients presented a change in *MGMT* promotor methylation.

In the initial tumor specimen, the *p15* promoter was methylated in 9 (22.5%) patients and non-methylated in 31 (85%) patients; in relapse tumors, these numbers were 5 (12.5%) and 34 (85%), respectively. Whilst the *p15* methylation site stayed stable between the initial and relapse tumors in 25 (62.5%) patients, instability was found in 14 (35%) cases.

The *p16* promoter in the initial tumor specimen was methylated in 10 (25%) cases. Another 30 (75%) cases harbored no methylation at this site. The corresponding relapse tumors revealed 5 (12.5%) cases with and 34 (85%) cases without *p16* methylation. Of the total collective, 24 (60%) patients presented with a stable *p16* promotor methylation and 15 (34.1%) patients with a change in *p16* promoter methylation.

**Figure 1:**
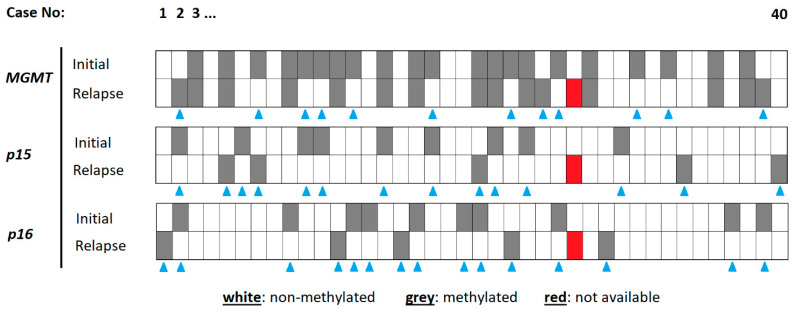
Details of methylation analysis for each specimen. The cases are organized from left to right (1–40). The different methylation sites are indicated on the left, for the initial tumor and the relapse tumor. A methylated gene promoter is indicated in grey, a non-methylated one in white. The blue triangles indicate cases with a chance in methylation status between initial and relapse tumor tissue. The red tiles represent cases with no methylation result.

The details of the methylation analysis are highlighted in Figure 1.

### 3.3. Stability of miRNA Expression

**Figure 2:**
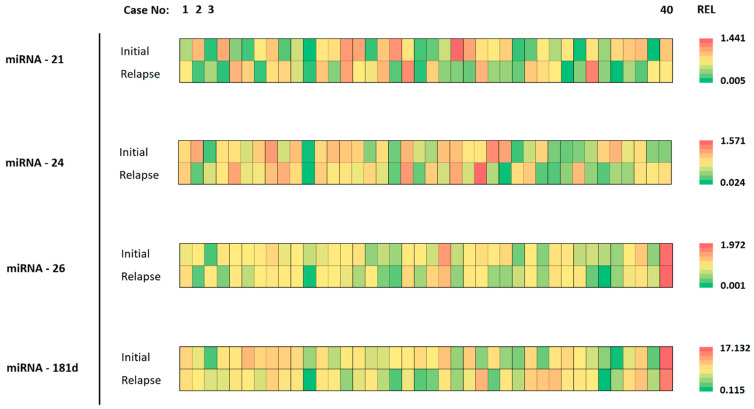
Details of miRNA analysis for each specimen. The cases are organized from left to right (1–40). The different analyzed miRNAs are shown on the left, for the initial tumor and the relapse tumor. The color of each tile represents the expression value for the respective tumor specimen. The scale of color ranges from green (lowest expression) to red (highest expression) for the indicated miRNA.

The expression of miRNAs in corresponding initial and relapse tumor specimens varied considerably. Individual cases presented with nearly the same miRNA expression level while others showed considerable heterogeneity between initial and relapse tumors. This is highlighted in Figure 2, where different colors represent the differing expression levels (REL).

**Figure 3:**
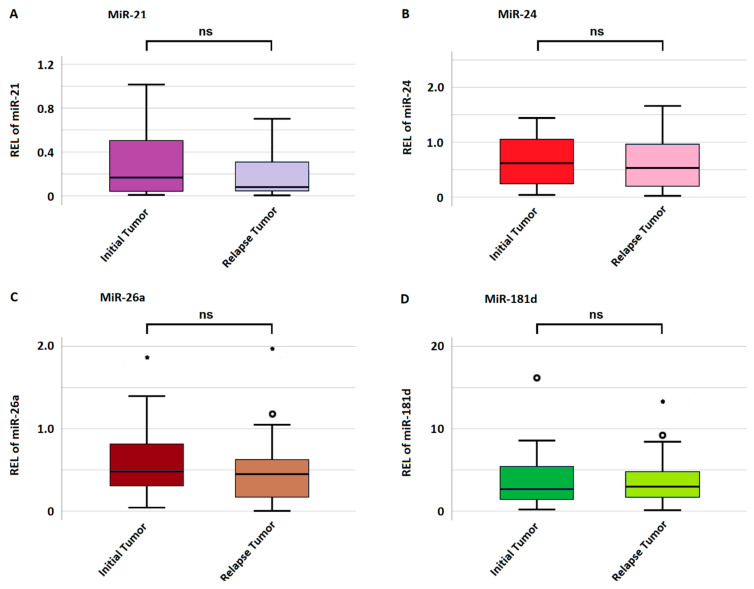
Mean miRNA expression in initial and relapse tumors. (**A**–**D**) indicate the expression of miRNA-21, miRNA-24, miRNA-26a, and miRNA-181d, respectively. The *y*-axis represents miRNA expression measured as relative expression level (REL). If a value is more than 1.5 standard deviations from the mean, it is considered a mild outlier and marked with a small circle (°). Extreme outliers, more than 3 standard deviations from the mean, are visualized with a star (*).

The mean miRNA expression in initial and relapse tumors was 0.29 ± 0.33 and 0.23 ± 0.28 for miRNA-21; 0.69 ± 0.61 and 0.62 ± 1.17 for miRNA-24; 0.65 ± 1.06 and 0.43 ± 0.56 for miRNA-26a; and 4.51 ± 4.43 and 3.21 ± 4.62 for miRNA-181d, respectively. There was no significant difference in miRNA expression between the initial and relapse tumors for any miRNA as highlighted in Figure 3. A gain in miRNA expression was considered if the relapse tumor harbored a value higher than the initial tumor and vice versa for a loss in expression. Regarding miRNA-21, 19 (47.5%) cases showed a gain and 21 (52.5%) a loss in expression. In regard to miRNA-24, 18 (45%) cases showed a gain, and 22 (55%) showed a loss in expression. Regarding miRNA-26a, 15 (37.5%) cases showed an increase, and 25 (62.5%) a decrease in expression. Lastly, regarding miRNA-181d, 15 (37.5%) cases showed a gain and 25 (62.5%) a loss in expression.

### 3.4. Impact of Epigenetic Temporal Stability on Survival

*MGMT* methylation in the initial tumor specimen was significantly associated with prolonged survival (*p* = 0.032). Patients with a methylated MGMT promoter had an overall survival of 21.2 ± 7.2; [1.5–60.6] months, those without 16.5 ± 8.3; [4.2–36.7] months. *MGMT* methylation in relapse tumors or change in methylation status did not impact survival. However, patients with a loss in miRNA-21 expression in relapse tumors survived significantly longer (*p* = 0.023). Patients with a loss in miRNA-21 expression survived 23.3 ± 8.7 [4–60.6] months; patients with a gain 14.9 ± 10 [1.5–41.2] months. In a multivariate ANOVA, this effect was independent of age of the patient at diagnosis, extent of resection, IDH mutation, and MGMT methylation. IDH mutation and MGMT methylation status were not correlated with miRNA-21 gain or losses. A comparable effect was demonstrated in an independent TCGA cohort, where patients with a decreased (below the median of the TCGA cohort) miRNA-21 expression presented with a significantly prolonged survival (*p* = 0.017).

**Figure 4:**
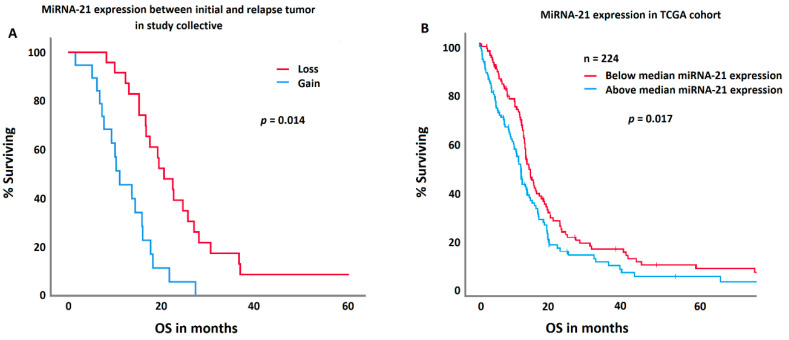
A comparison between the study collective (**A**) and the TCGA control group (**B)** regarding miRNA-21 expression and survival. Patients with a decrease in the miRNA-21 expression in their relapse tumor (red curve) showed a significantly longer survival in both cohorts.

In comparison, neither the methylation statuses of *p15* and *p16* nor the expression of miRNA-24, -26a, or -181d had any significant impact on survival or the course of the disease. This is highlighted in Figure 4

## 4. Discussion

Epigenetic tumor characteristics have come more and more into focus as tools for the prediction of glioblastoma prognosis. The aim of the study at hand was to evaluate the stability of specific miRNA expressions and the methylation statuses of *MGMT*, *p15*, and *p16* between primary tumors and relapse tumors, whilst also exploring if the dynamics of the aforementioned characteristics influences survival.

### 4.1. Temporal Stability of Epigenetic Markers

The study at hand indicates that a change in promoter methylation of *MGMT*, *p15* and *p16* between initial and relapse glioblastoma occurs in roughly one-third of the cohort. Recent studies have already addressed the dynamic of *MGMT* methylation in glioblastoma progression. O`Regan et al. measured a 36.4% rate of instability between initial and relapse tumors in 22 glioblastoma patients [24]. The MGMT methylation status was acquired via pyrosequencing. Brandes et al. highlighted an *MGMT* instability rate of 25% in a larger cohort with 108 patients. In their study, methylation-specific PCR was used as an analytical tool [25]. A comprehensive review of the literature by Feldheim et al. highlighted a change in *MGMT* methylation in 24.2% of the cases in a cohort of 476 patients. A gain or loss in promoter methylation occurred comparably often [36]. These reports in the literature resemble the data of the study at hand regarding *MGMT* methylation.

In addition to this, *p15* and *p16* methylation status was acquired for the present study. The rates of change between initial and relapse tumors were comparable to that of *MGMT*. The literature here is scarcer, as most studies focus on *MGMT* in glioblastoma, due to its known impact on therapy response to temozolomide. A case report of a 30-year-old glioblastoma patient highlighted a change in over 10,000 methylation sites throughout the genome between initial and recurrent tumor specimens; however, *p15* and *p16* were not considered in this report [37]. This, altogether, shows that a change in various methylation sites may occur in a considerable portion of the cases. The reason, therefore, is not fully understood. Some authors ascribe these dynamics to arbitrary coincidence, while others point out that chemotherapy with alkylating agents like temozolomide may cause these changes [38,39].

The present trial shows considerable variations in the expression of miRNA-21, -24, -26a, and 181d between each initial and relapse tumor. Data from the literature for the dynamics of miRNA expression during the progression of GBM are even more limited than those of the dynamics of the methylation status of various gene promoter sites. A single case analysis of a recurrent GBM by Park et al. studied the expressions of 318 miRNAs. The expression of 43 of those miRNAs was significantly altered in primary and recurrent tumors. In their study, microarray analysis and RT-qPCR were used as analytic tools [40]. Next to this case report, to the best of the knowledge of the authors, there is no other study available searching for those miRNA dynamics in a cohort of comparable size to that at hand.

At this point, the underlying mechanism for these changes in miRNA expression remains unclear. Whether this is due to a change in the actual epigenetic characteristics between the onset of the primary or relapse disease, or maybe caused by the therapy used as some authors propose, or due to the huge heterogeneity of the tumor, is not fully understood at this point [41]. Nevertheless, can the long-known characteristics of heterogeneity in individual GBM also be ascribed on an epigenetic level, including miRNA expression? Interestingly, no change in the mean expression of the studied miRNAs was observed in the total collective.

### 4.2. Influences of Temporal Instability of Epigenetic Markers on the Survival of GBM Patients

The previous literature shows the important role of miRNA-21, -24, -26a, and -181d in the prognosis of GBM [29,33,42]. In the study at hand, we show that patients with a decreased expression of miRNA-21 had significantly longer survival than patients with a stable or increased expression of said miRNA. This supports the existing literature in which miRNA-21 is described as an onco-miRNA. It influences important pathways like insulin-like growth factor (IGF)-binding protein -3 (IGFBP3) or RECK, and TIMP3 [31]. Patients with a high expression of miRNA-21 show shorter survival times, while patients with a low expression survive longer [42]. A major point how this effect of miRNA-21 on glioblastoma might be mediated is the initially mentioned P15/P16/RBI/E2F pathway, especially RB1, with is a direct target of miRNA-21. By inhibition of the tumor suppressor RB1, and, therefore, a subsequent non-inhibition of the cell cycle, gliomagenesis might be co-triggered [43]. This, in combination with our findings, supports the important role of miRNA-21 in glioblastoma pathogenesis and supports the aforementioned theory that changes in miRNA expressions provide information on the malignancy of the tumor. None of the other miRNAs had an effect on the survival of GBM patients. The change in methylation status of *MGMT*, *p15*, and *p16* also did not influence survival. Regarding miRNA-26a and -181d, this comes as no surprise, since they only show an influence on survival in patients treated with carmustine wafers [29,44]. It needs to be acknowledged that due to the low numbers in sub-cohorts of patients with a gain or loss of miRNA expression, the conclusions drawn have to be interpreted carefully.

The effect of the use of miRNAs as liquid biopsy markers should also be considered. The study at hand shows significant differences between miRNAs in primary and recurrent tumors, which could be useful in the early detection of relapses. As our group could show, there is a correlation between the miRNA expression in tumor tissue and blood [34].

### 4.3. Limitations of the Study

The main limitation of the present study is the small number of included patients. It remains a challenge to collect huge number of patients who underwent multiple GBM resections and fitting for inclusion criteria. Therefore, the results at hand have to be scrutinized carefully. Furthermore, it remains unclear whether the change in miRNA expression between primary and recurrent tumors resembles a change in the epigenetic of the tumor itself or is a result of its heterogeneity within individual tumors.

## 5. Conclusions and Further Perspective

The data demonstrate that changes in miRNA-21 expression between the primary tumor and the relapse tumor have a significant influence on the survival of glioblastoma patients. This suggests the possibility of using miRNA expression changes between the onset of the primary and relapse tumors as a prognostic tool. Hence, these changes should be further evaluated in bigger cohorts. Future studies in which the miRNA expression in the blood is studied over the course of the disease could answer the question of whether certain miRNAs are reliable recurrence detection markers.

## Figures and Tables

**Table 1:** Patient characteristics and treatment details at first diagnosis.

Characteristic	All Patients with GBM (*n* = 44)
Mean age ± SD [range] (years)	56.9 ± 9.5 [35.9–77.9]
Sex, *n* (%)	
Male	27 (61.4)
Female	17 (38.6)
Extent of resection	
Gross total resection	21 (47.7)
Subtotal resection	23 (52.3)
Overall survival ± SD [range] (month)	19.5 ± 10.8 [1.5–60.6]
Progression-free survival ± SD [range] (month)	9.9 ± 5.6 [0.9–23.4]
Death by end of trial, *n* (%)	42 (95.5)

## Data Availability

The used tested battery and analyzed data during the current study are available from the corresponding author upon reasonable request.
